# Epidemiology and Treatment Outcomes of Pulmonary Tuberculosis in Dazu District, Chongqing, China, 2005-2024: Surveillance Study

**DOI:** 10.2196/78564

**Published:** 2025-11-28

**Authors:** Yu Yu, Xiu-juan Hu, Xiao-man Fang, Jing Wu

**Affiliations:** 1 Infectious Department, The People's Hospital of Dazu, No.1073, Erhuan South Road, Tangxiang Subdistrict, Dazu District, Chongqing, 402360, China, 86 13648370220; 2 Research Department, The People's Hospital of Dazu, Chongqing, China

**Keywords:** China, Chongqing, Dazu District, epidemiology, pulmonary tuberculosis, treatment outcome, tuberculosis

## Abstract

**Background:**

As a high tuberculosis (TB) burden area in China, Dazu District of Chongqing Municipality contains a large rural population and exhibits typical features of TB endemicity.

**Objective:**

This study aimed to analyze the epidemiological characteristics and treatment outcomes of pulmonary tuberculosis (PTB) in Dazu District from 2005 to 2024, with the aim of supporting the optimization of regional TB control strategies.

**Methods:**

Data on PTB cases in Dazu District from 2005 to 2024 were collected from the China Disease Control and Prevention Information System. Descriptive epidemiological methods were employed to analyze the temporal, demographic, and geographical distributions, along with trends in treatment outcomes. Global and local spatial autocorrelation analyses were performed using Moran *I* and Getis-Ord Gi* statistics, respectively.

**Results:**

A total of 10,236 cases were reported, for an average annual notification incidence of 65.2 per 100,000 population. The annual average notification incidence decline rate was 7.7%. Joinpoint regression analysis revealed a statistically significant decline in annual incidence rates (average annual percent change=−6.81, 95% CI −7.25 to −6.30, *P*<.0001). The bacteriological positivity rate initially decreased before rising, reaching 81.6% in 2024. Reported case counts peaked in March, while relatively lower numbers were observed during October, November, and December. Cases were predominantly among male patients, with a male-to-female ratio of 3.57:1. The case composition ratio in the ≥65 years age group has gradually increased, from 13.8% in 2006 to 19.9% in 2015 and to 38.5% in 2024. Occupational distribution was primarily among farmers (77.6%, 7948/10,236), homemakers or unemployed individuals (5.6%, 570/10,236), and students (3%, 303/10,236). Cases were concentrated in Longshui Town (13.1%, 1339/10,236), Tangxiang Subdistrict (9.1%, 933/10,236), and Longgang Subdistrict (7.9%, 811/10,236)—areas with large population bases. Among these, Guoliang Town exhibited the highest average annual notification incidence (314.4/100,000). Treatment success rate reached 91.3%. Multivariate binary logistic regression revealed that age 25‐44 years (OR 1.755; 95% CI 1.320‐2.332; *P*<.0001), undergoing initial treatment (OR 3.786; 95% CI 2.524‐5.680; *P*<.0001), absence of HIV coinfection (OR 2.499; 95% CI 1.714‐3.643; *P*<.0001), negative bacteriologic test results (OR 2.841; 95% CI 2.214‐3.646; *P*<.0001), and the receipt of full-course supervised treatment (OR 7.705; 95% CI 4.520‐13.137; *P*<.0001) were significantly associated with treatment success.

**Conclusions:**

The notification incidence of PTB in Dazu District, Chongqing, has gradually declined. Particular focus is required on the treatment of young children, elderly individuals, patients with HIV coinfection, those under intensive phase supervision, bacteriologically positive cases, and retreatment cases. These measures may reduce the incidence of PTB and improve treatment success rates in our district.

## Introduction

Pulmonary tuberculosis (PTB), caused by *Mycobacterium tuberculosis* (*MTB*), is a chronic infection disease primarily affecting the lungs and represents the most common form of TB. As an airborne pathogen, *MTB* poses a serious global public health threat [1]. Globally, TB remains the leading cause of death from a single infectious agent [2]. According to the World Health Organization (WHO) Global Tuberculosis Report 2024 [3], there were approximately 10.8 million new TB cases and 1.25 million deaths in 2023. In response, the WHO and the United Nations have set a goal to reduce TB deaths by 95% and new cases by 90% by 2035. China bears one of the highest TB burdens globally, reporting 741,000 new cases in 2023—an estimated incidence of 52 per 100,000 population. It ranks third among the 30 high-TB burden countries, accounting for 6.8% of the global cases. An estimated 25,000 TB-related deaths occurred, corresponding to a mortality rate of 2 per 100,000. The treatment success rate for drug-sensitive TB is approximately 88%. These figures indicate that China faces significant challenges in meeting the WHO’s goal to end the TB epidemic by 2035.

The prevalence of TB exhibits notable spatial heterogeneity and demographic disparities. In China, regions with higher TB burdens are often associated with factors such as economic development, health care accessibility, and population mobility. Previous studies have indicated higher incidence rates in western regions and rural areas, with farmers, older adults, and male individuals being disproportionately affected [4-7]. As a municipality in western China with both extensive urban and rural populations, Chongqing plays a critical role in regional TB control efforts. However, compared with national or provincial macro-level data, long-term dynamic monitoring studies at the district and county level remain relatively scarce. Although several studies at the urban and county scale in Chongqing have been published [8-11], they are often limited by short time spans, small sample sizes, and insufficient depth.

District- and county-level dynamic surveillance of TB is critical for elucidating local transmission dynamics and evaluating the effectiveness of disease control interventions. In this study, we leverage 2 decades (2005‐2024) of surveillance data from Dazu District, Chongqing, to track temporal trends with Joinpoint regression, map spatial hotspots through geospatial techniques, and identify key epidemiological and treatment-related factors. This study aims to provide a scientific basis for optimizing local TB control strategies and advancing the goal of TB elimination by elucidating the epidemiological characteristics and temporal dynamics of PTB at the district and county levels.

## Methods

### Data Sources

Patient data of PTB cases from Dazu District, Chongqing, with confirmed diagnosis dates between January 1, 2005, and December 31, 2024 were collected from the Tuberculosis Management Information System (TBIMS) subsystem of the China Information System for Disease Control and Prevention (retrieved 2025-03-28). Data fields included current address, gender, age, registration date, bacteriological results, population category, key populations, symptom onset date, initial consultation date, complications, diagnosis confirmation date, treatment initiation date, and reasons for treatment termination. The exclusion criteria comprised duplicate reports, errors in basic infectious disease report card information, missing essential data, diagnostic modifications, cases initially managed outside Dazu District, extrapulmonary TB, untreated cases, and nontuberculous mycobacterial infections.

Case management data were retrieved from the TBIMS, with initial registration management units being the Dazu District Tuberculosis Prevention and Control Center or Dazu District People’s Hospital of Chongqing (designated medical institution). The TBIMS was launched in 2005 using paper-based reporting and manual entry and was upgraded to a web-based real-time electronic system in 2010, greatly reducing latency. It covers all TB control institutions across 31 provinces, over 300 prefectures, and 3000 counties in mainland China, mandating reporting for all bacteriologically confirmed or clinically diagnosed TB cases. Now the world’s largest TB registration system, the TBIMS, is recommended by the WHO as a model for global TB surveillance.

This study analyzed incidence characteristics (temporal, geographical, and demographic distributions) and investigated influencing factors of treatment outcomes. Annual notification rates used end-of-year permanent-resident denominators from the *Dazu District Statistical Yearbooks* published by Dazu District, Chongqing, during 2005-2024 (retrieved 2025-03-31). Age-standardized incidence rates were calculated using the 2020 Seventh National Population Census data (National Bureau of Statistics) as the standard population. The names of the 27 townships or streets involved in this study are all standardized administrative division units, which do not reflect the differences in special geographical or social attributes. Its division is based on the “Chongqing Urban and County Administrative Division Code” (GB/T 2260), which only reflects the administrative management level.

### Research Methods

#### Diagnosis and Treatment of PTB

Patients were diagnosed according to the 《*WS 288‐2008 Pulmonary Tuberculosis Diagnostic Criteria*》, 《*WS 288‐2017 Pulmonary Tuberculosis Diagnosis* 》 [12,13], and 《*WS 196‐2017 Tuberculosis Classification*》 [14] standards. All TB treatments were implemented in accordance with the 《*Technical Guidelines for Tuberculosis Prevention and Control in China (2021 Edition)》* [15]. To maintain data comparability across the study period, TB cases in this research included tuberculous pleuritis cases throughout all calendar years.

#### Definitions

Definitions are as follows: (1) case detection methods: referral, active screening, direct consultation or referral, contact tracing, health examination, and others (cases with undetermined or unclassifiable sources). (2) Discovery delay: the total time interval between a patient’s initial clinical symptom and confirmed TB diagnosis, encompassing both care-seeking and diagnostic confirmation delays. This study employed a 28-day threshold as the criterion for determining discovery delay. (3) Bacteriological results: from 2005 to 2018, smear-positive or culture-positive cases were classified as bacteriologically positive, while cases with negative results in both smear microscopy and culture were classified as bacteriologically negative. From 2019 to 2024, cases with any positive result in smear microscopy, culture, or molecular biological testing were classified as bacteriologically positive, whereas cases with negative results in all 3 methods were classified as bacteriologically negative. Patients with tuberculous pleurisy were uniformly considered bacteriologically negative. (4) Number of patients with PTB: it refers to cases registered and reported between 2005 and 2024 with diagnostic confirmation of TB (including clinical diagnoses) and including patients with tuberculous pleurisy. (5) Treatment outcome determination: according to standards established in the 《*Technical Specifications for Tuberculosis Prevention and Control in China (2020 Edition*) 》 [16], treatment outcomes are categorized into either treatment success (including cure and treatment completion) or unfavorable outcome (including mortality, loss to follow-up, treatment discontinuation, and revision of therapy to a multidrug-resistant TB regimen).

#### Observation Indicators

Observation indicators are as follows: (1) notification incidence rate: it refers to the proportion of patients with registered and reported PTB to the total population within a specified period. (2) Age-standardized incidence rate: it is an incidence rate calculated by weighting age-specific rates of a study population to a standard population structure, enabling comparisons unaffected by differences in age distribution. (3) Calculation formula for average annual decline rate of PTB notification incidence rate: average annual decline rate=(1 - $\sqrt[{n}]{P_{n}/P_{0}}$)×100%, where P_n_ represents the registered incidence rate in the nth year, P₀ denotes the registered incidence rate in the base year, and n indicates the number of interval years. (4) Successful treatment rate = (Cured+Treatment completion)/(Number of pulmonary tuberculosis patients–Transferred to rifampicin-resistant treatment–Diagnosis changed)×100%.

### Ethical Considerations

This study was submitted to the Ethics Commission of the People’s Hospital of Dazu District. This study does not involve human participants or animals. Ethics approval was not required for this study because we did not include any identifiable data of patients’ personal information, including name, identity information, address, and telephone number. This study reviewed only secondary aggregated data at the population level; therefore, the need for written informed consent was waived. The Ethics Committee of the People’s Hospital of Dazu District approved this secondary analysis of deidentified TBIMS data (Approval 2025LLSC158; 2025-03-12) and granted a waiver of informed consent.

### Statistical Analysis

#### Descriptive Analysis

Data were organized, and a database was established using Microsoft Excel 2021. Statistical analysis was performed using SPSS (version 27.0; IBM SPSS Statistics,) and Joinpoint (version 5.2.0; National Cancer Institute of America) software. Descriptive epidemiologic methods were employed. Count data were described using “number of cases, incidence rate (/100,000), and composition ratio (%).“ Intergroup comparisons were analyzed using the *χ*² test.

#### Joinpoint Regression

The temporal trend change points in age-standardized incidence rates were identified using the Joinpoint Regression Program (version 5.40; National Cancer Institute of America). This method fits the natural logarithm of the incidence rate as a piecewise linear function. The optimal number of joinpoints was determined and adopted using a Monte Carlo permutation test (10,000 iterations, *α*=.05). The independent variable was the calendar year, the interval type was set to annual, and the data point offset was 0. The dependent variables were the annual notification incidence rate and the age-standardized notification incidence rate, with data points where the incidence rate was 0 being excluded. The annual percent change (APC) along with its 95% CI was calculated for each trend segment, and the average annual percent change (AAPC) for the entire study period was also computed. The overall significance level for the permutation test was set at 0.0500. The maximum number of joinpoints was constrained to 3 based on the 19-year observation period, and model selection was optimized using the Bayesian information criterion.

#### Spatial Analysis

Global and local spatial autocorrelation analyses were performed using Moran *I* and Getis-Ord Gi* statistics, respectively. The Moran *I* index ranges from −1 to 1, where values approaching +1 indicate positive spatial clustering (similar values cluster together), values near 0 suggest a random distribution, and values approaching −1 indicate dispersion. Statistically significant Giclusters were classified as hotspots (high-value clusters; *P*<.05, |*Z*|>1.96) or coldspots (low-value clusters; *P*<.05, |*Z*|<1.96).

#### Logistic Regression

A binary multivariate logistic regression analysis model was used to analyze the influencing factors of treatment outcomes; a *P*<.05 was considered statistically significant.

## Results

### Reported Incident Case Count, Notification Incidence Rates, and Bacteriological Positivity Rates

**Figure 1.**
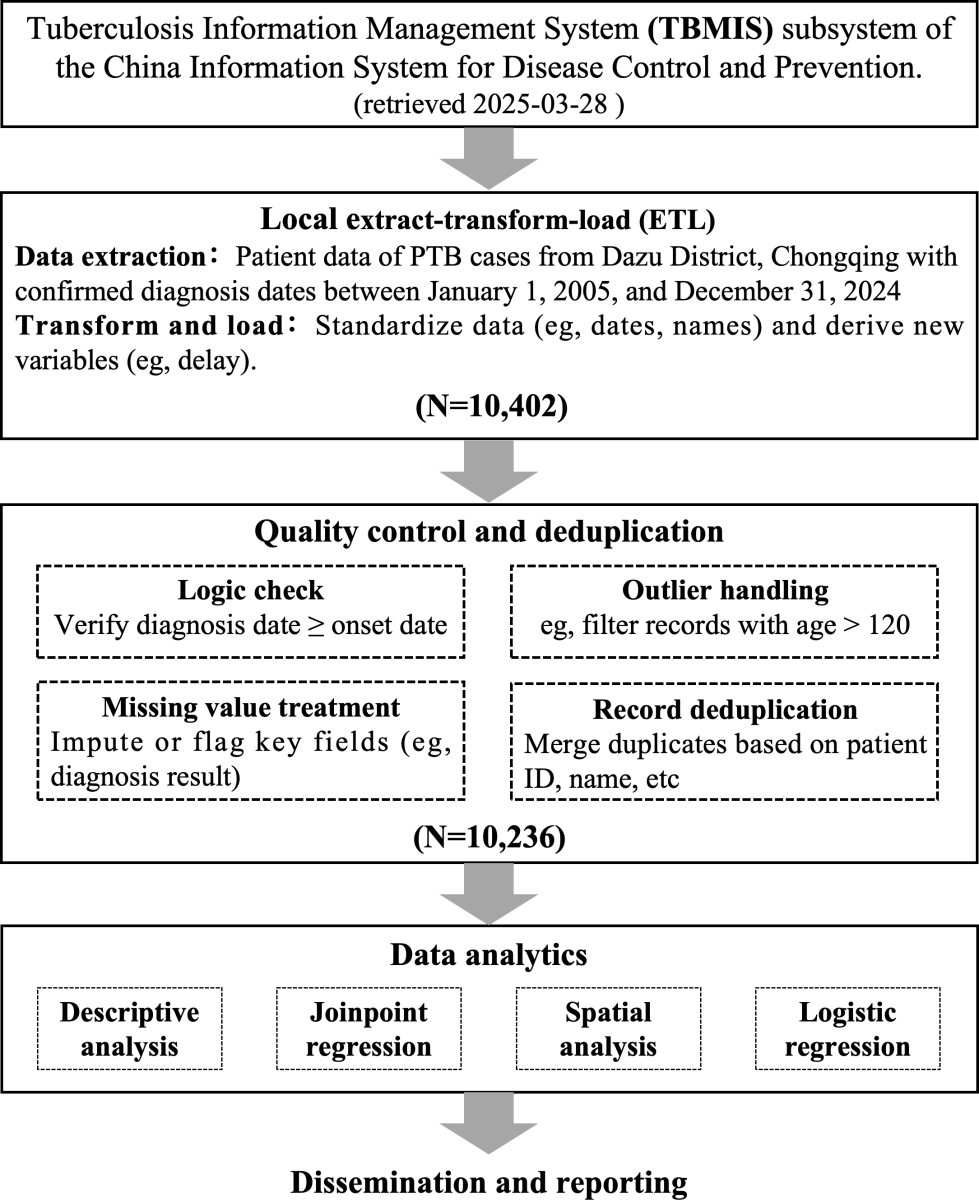
Data flow diagram. PTB: pulmonary tuberculosis.

All regions share the same case reporting system and diagnostic criteria (Figure 1 and Checklist 1). A total of 10,236 cases were reported. The highest reported case count was in 2005 with 1000 cases, followed by a gradual downward trend, reaching a nadir of 234 cases in 2024. The notification incidence similarly decreased from 131.2/100,000 in 2005 to 28.4/100,000 in 2024, with an average annual notification incidence of 65.2/100,000. The average annual decline rate of reported TB incidence was 7.7%. The bacteriological positivity rate gradually declined from 46.9% in 2005 to 31.6% in 2017 and then subsequently increased to 81.6% by 2024. Monthly analysis revealed that March had the highest cumulative reported case count (1098 cases), while October, November, and December showed relatively lower counts, with 720, 750, and 748 cases, respectively (Tables1  2).

**Table 1.** Reported incident case count, annual and age-standardized incidence rates, and bacteriological positivity rates of pulmonary tuberculosis in Dazu District, Chongqing, China, 2005‐2024.

Year	Permanent resident population (100,000 persons)	Reported cases (n)		Bacteriological positivity (%)	Annual notification incidence rate (per 100,000 population)	Age-standardized incidence rate (per 100,000 population)		
		Reported case count	Bacteriologically positive case count			<15	15‐64	≥65
2005	76.2	1000	469	46.9	131.2	2.6	15.8	13.4
2006	76.0	701	294	41.9	92.2	1.0	11.1	10.8
2007	76.0	810	373	46.0	106.6	0.9	12.3	15.3
2008	76.7	747	365	48.9	97.4	0.6	11.5	12.8
2009	77.0	663	356	53.7	86.1	0.4	10.1	11.8
2010	67.1	630	346	54.9	93.9	0.0	11.1	13.2
2011	73.6	508	156	30.7	69	0.3	8.2	9.3
2012	74.9	594	73	12.3	79.3	0.4	9.1	12.0
2013	76.1	587	134	22.9	77.1	0.2	9.0	11.0
2014	77.2	554	103	18.6	71.8	0.1	8.6	9.3
2015	78.4	534	118	22.1	68.1	0.1	7.9	10.0
2016	80.0	508	124	24.4	63.5	0.2	7.3	9.6
2017	80.5	351	111	31.6	43.6	0.1	5.1	6.3
2018	81.9	357	151	42.3	43.6	0.1	4.8	7.7
2019	82.7	347	175	57.8	42	0.2	4.6	7.4
2020	83.6	285	149	52.3	34.1	0.1	3.7	6.5
2021	83.6	305	177	58.0	36.5	0.1	3.6	8.7
2022	83.4	255	168	65.9	30.6	0.1	2.8	8.4
2023	82.1	266	177	66.5	32.4	0.1	2.9	8.9
2024	82.3	234	191	81.6	28.4	0.0	2.6	8.1

**Table 2.** Monthly cumulative reported tuberculosis (TB) cases in Dazu District, Chongqing, China, 2005-2024.

Year	Month (n)												Annual total cases (n)
	January	February	March	April	May	June	July	August	September	October	November	December	
2005	70	65	141	111	73	91	63	84	80	54	84	84	1000
2006	84	93	85	75	42	51	42	51	46	42	46	44	701
2007	61	50	81	76	77	81	74	75	59	64	61	51	810
2008	48	68	87	59	67	56	46	80	74	56	42	64	747
2009	30	66	80	64	68	74	60	36	47	40	43	55	663
2010	61	41	69	48	44	67	67	45	55	42	50	41	630
2011	38	39	40	43	44	44	49	44	54	42	41	30	508
2012	40	63	76	54	56	59	48	48	42	38	45	25	594
2013	43	41	74	58	49	45	50	60	57	31	46	33	587
2014	42	53	47	55	49	54	49	41	63	40	30	31	554
2015	35	39	45	39	37	52	48	50	30	53	49	57	534
2016	30	43	63	46	43	52	37	35	42	46	36	35	508
2017	34	55	42	24	28	24	19	25	20	30	24	26	351
2018	31	26	26	31	42	28	35	30	33	27	10	38	357
2019	27	30	38	27	26	32	33	7	61	8	32	26	347
2020	5	0	6	68	39	19	19	26	30	21	25	27	285
2021	26	14	31	22	28	28	24	27	34	22	14	35	305
2022	29	14	22	22	26	18	22	33	18	19	22	10	255
2023	19	26	19	24	24	19	27	31	20	24	21	12	266
2024	14	14	26	11	16	20	15	18	26	21	29	24	234
Monthly total cases (n)	767	840	1098	957	878	914	827	846	891	720	750	748	10,236

**Figure 2.**
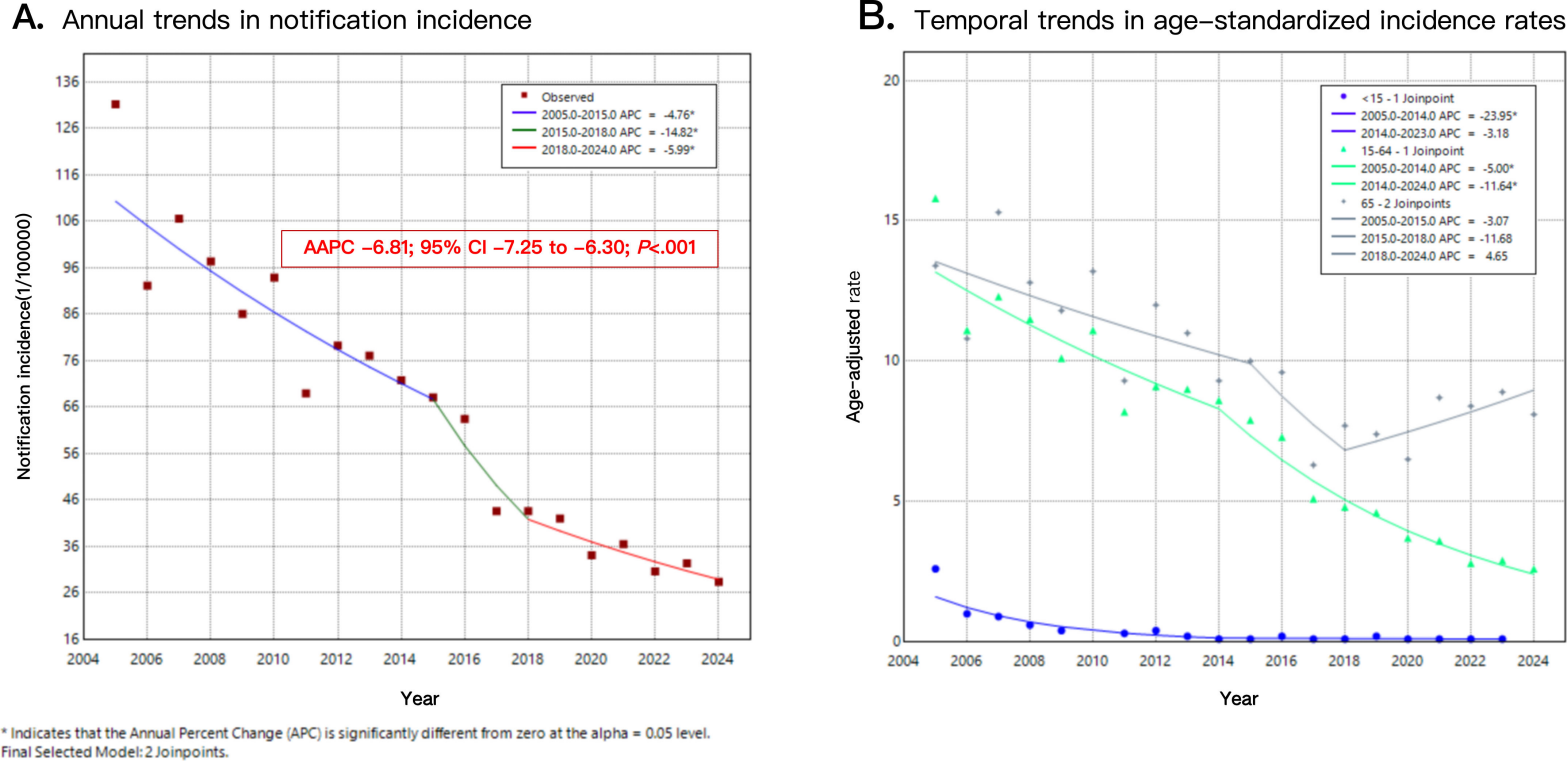
Trend chart of annual notification pulmonary tuberculosis (PTB) incidence in Dazu District, Chongqing, China, 2005-2024: Joinpoint regression.

The Joinpoint regression analysis revealed that the annual notification incidence of PTB in Dazu District exhibited a significant overall downward trend (AAPC=−6.81; 95% CI −7.25 to −6.30; *P*<.001). This overall trend was characterized by a fluctuating decline across 3 distinct phases: a slight decrease from 2005 to 2015 (APC=−4.76; 95% CI −5.58 to −3.63; *P*=.002), an accelerated decline from 2015 to 2018 (APC=−14.82; 95% CI −16.96 to −10.04; *P*=.002), followed by a moderated rate of decline from 2018 to 2024 (APC=−5.99; 95% CI −7.83 to −0.38; *P*=.04; Figure 2A).

The trends in age-standardized notification incidence of PTB in Dazu District varied across different age groups. The <15 years age group experienced a significant initial decline (2005‐2014: APC=−23.95; 95% CI −37.04 to −18.47; *P*<.001), which then plateaued (2014‐2023: APC=−3.18; 95% CI −9.89 to 13.39; *P*=.48). The 15‐64 years age group showed the largest overall decrease, with an accelerating downward trend (2005‐2014: APC=−5.00; 95% CI −6.51 to −2.73; *P*=.004; 2014‐2024: APC=−11.64; 95% CI −13.47 to −10.36; *P*<.001). In contrast, the ≥65 years age group, after a period of fluctuating decline (2005‐2015: APC=−3.07; 95% CI −5.32 to −10.80; *P*=.14; 2015‐2018: APC=−11.68; 95% CI −17.20 to 0.15; *P*=.05), transitioned to a slow increase (2018‐2024: APC=4.65; 95% CI −1.78 to 18.40; *P*=.08; Figure 2B).

### Demographic Distribution

**Figure 3.**
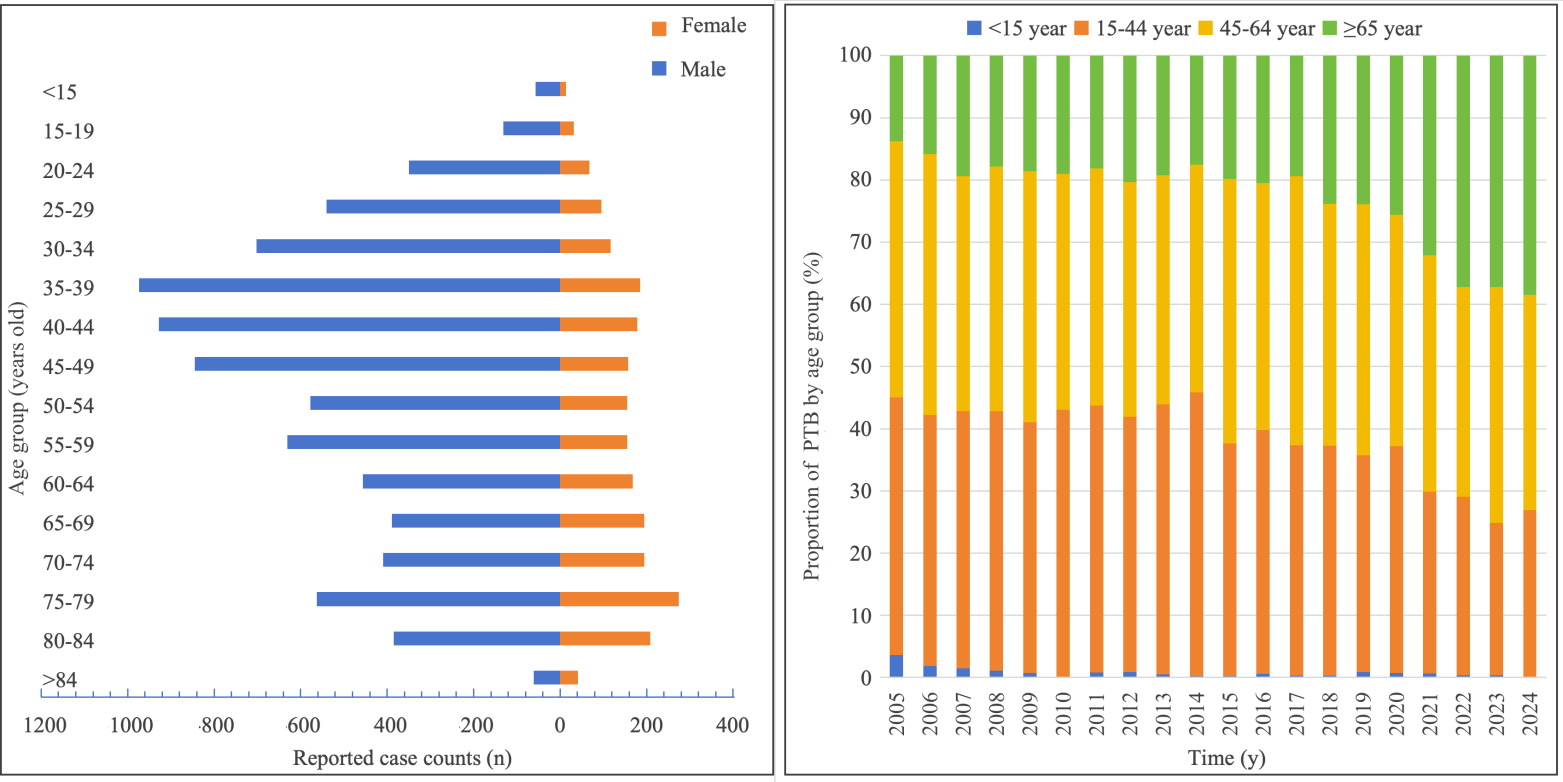
Reported case counts and proportions of pulmonary tuberculosis (PTB) across age groups by year in Dazu District, Chongqing, China, 2005-2024.

Reported patients comprised 7998 cases among male patients and 2238 cases among female patients, yielding a male-to-female ratio of 3.57:1. When categorized in 5-year intervals, male and female cases predominantly occurred in the 15‐64 age group, with relatively uniform case distribution across subintervals within this range (Figure 3). Throughout the observation period and across all populations, children <15 years accounted for 102 cases (0.9%), young adults aged 15‐44 years comprised 4028 cases (39.4%), middle-aged adults 45‐64 years constituted 4000 cases (39.1%), and older patients ≥65 years represented 2106 cases (20.6%). The proportion of patients ≥65 years showed a gradually increasing trend, while the incidence proportion among young adults 15‐44 years progressively declined. The proportion among middle-aged adults 45‐64 years remained stable (Table 3, Figure 3).

**Table 3.** Reported case counts and percentages of pulmonary tuberculosis (PTB) across age groups in Dazu District, Chongqing, 2005 to 2024.

Year	Age group (y), n (%)			
	<15	15‐44	45‐64	≥65
2005	36 (3.6)	414 (41.4)	412 (41.2)	138 (13.8)
2006	13 (1.8)	283 (40.4)	294 (41.9)	111 (15.8)
2007	12 (1.5)	335 (41.4)	306 (37.8)	157 (19.4)
2008	8 (1.1)	312 (41.8)	294 (39.4)	133 (17.8)
2009	5 (0.8)	267 (40.3)	268 (40.4)	123 (18.6)
2010	0 (0)	271 (43)	239 (37.9)	120 (19.1)
2011	4 (0.8)	218 (42.9)	194 (38.2)	92 (18.1)
2012	5 (0.8)	244 (41.1)	224 (37.7)	121 (20.4)
2013	3 (0.5)	255 (43.4)	216 (36.8)	113 (19.3)
2014	1 (0.2)	253 (45.7)	203 (36.6)	97 (17.5)
2015	1 (0.2)	200 (37.5)	227 (42.5)	106 (19.9)
2016	3 (0.6)	199 (39.2)	202 (39.8)	104 (20.5)
2017	1 (0.3)	130 (37)	152 (43.3)	68 (19.4)
2018	1 (0.3)	132 (37)	139 (38.9)	85 (23.8)
2019	3 (0.9)	121 (34.9)	140 (40.3)	83 (23.9)
2020	2 (0.7)	104 (36.5)	106 (37.2)	73 (25.6)
2021	2 (0.7)	89 (29.2)	116 (38.0)	98 (32.1)
2022	1 (0.4)	73 (28.6)	86 (33.7)	95 (37.3)
2023	1 (0.4)	65 (24.4)	101 (38)	99 (37.2)
2024	0 (0)	63 (26.9)	81 (34.6)	90 (38.5)

The top 3 occupational groups were farmers (77.6%, 7948/10,236), homemakers or unemployed individuals (5.6%, 570/10,236), and students (5.2%, 532/10,236), accounting for 88.4% (9050/10,236) of the total reported cases. These were followed by workers and migrant workers (3.0%, 303/10,236), commercial service personnel (1.1%, 116/10,236), and government employees (0.9%, 93/10,236). The remaining occupations accounted for less than 7% collectively (Multimedia Appendix 1).

### Geographical Distribution

All towns or subdistricts in Dazu District except for Longtanzi Subdistrict reported cases annually. The highest reported case count was in Longshui Town (13.1%, 1339/10,236), followed by Longgang Subdistrict (9.1%, 933/10,236) and Tangxiang Subdistrict (7.9%, 811/10,236). The lowest case count occurred in Longtanzi Subdistrict (0.7%, 72/10,236).

**Figure 4.**
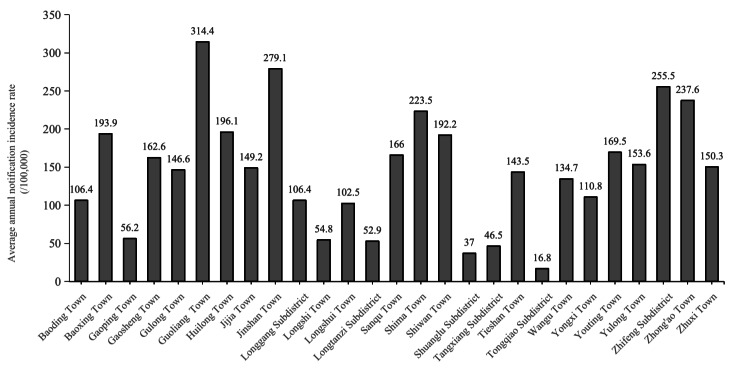
Average annual notification incidence rate of pulmonary tuberculosis (PTB), Dazu District, Chongqing, China, 2005-2024.

**Figure 5.**
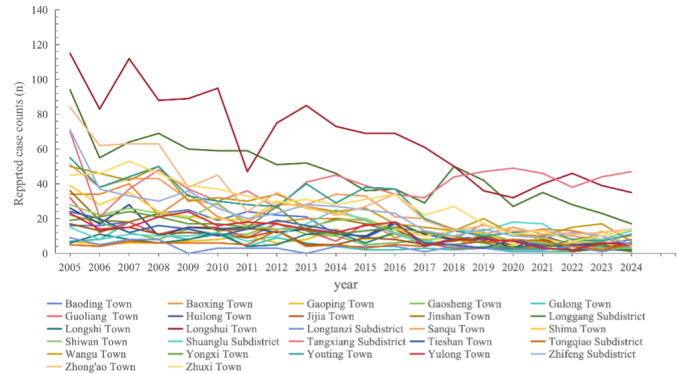
Trends in reported tuberculosis (TB) case counts by town or subdistrict and year in Dazu District, Chongqing, China.

Due to the difficulty in obtaining accurate annual resident population data for each town and street, the proportion of reported cases in each town relative to the district’s total annual cases was used as a substitute for the annual incidence rate (Table 4 and Figure 4). Longshui Town had the highest proportion of the reported cases in most years, exceeding 10% (47/234) in all years except 2011, although its case numbers showed a clear downward trend; it was followed by Longgang Street. In Tangxiang Street, the annual case count declined from 2005 to 2017 and then gradually fluctuated upward. By 2024, it had the highest number of reported cases in the district, accounting for 20.1% (Table 5, Figures5  6).

**Table 4.** Annual pulmonary tuberculosis (PTB) average annual notification incidence rates by town or subdistrict in Dazu District.

Region	Average annual notification incidence rate (/100,000)	Resident population	Total case count (n)	Average annual case count (/100,000)
Baoding Town	106.4	15,037	274	13.7
Baoxing Town	193.9	17,534	375	18.7
Gaoping Town	56.2	12,451	140	7.0
Gaosheng Town	162.6	13,534	171	8.55
Gulong Town	146.6	4774	107	5.35
Guoliang Town	314.4	10,178	209	10.4
Huilong Town	196.1	13,260	243	12.1
Jijia Town	149.2	11,392	169	8.4
Jinshan Town	279.1	12,900	268	13.4
Longgang Subdistrict	106.4	88,330	933	46.6
Longshi Town	54.8	10,952	130	6.5
Longshui Town	102.5	1,12,170	1339	66.9
Longtanzi Subdistrict	52.9	17,023	72	3.6
Sanqu Town	166.0	30,722	495	24.7
Shima Town	223.5	17,452	370	18.5
Shiwan Town	192.2	14,565	290	14.5
Shuanglu Subdistrict	37.0	40,575	250	12.5
Tangxiang Subdistrict	46.5	1,50,488	811	40.5
Tieshan Town	143.5	16,721	227	11.3
Tongqiao Subdistrict	16.8	29,758	113	5.6
Wangu Town	134.7	37,108	516	25.8
Yongxi Town	110.8	17,154	270	13.5
Youting Town	169.5	32,443	543	27.15
Yulong Town	153.6	14,978	251	12.5
Zhifeng Subdistrict	255.5	27,786	462	23.1
Zhong’ao Town	237.6	35,360	635	31.7
Zhuxi Town	150.3	29,947	573	28.6

**Table 5.** Annual pulmonary tuberculosis (PTB) incident case counts by town or subdistrict in Dazu District, Chongqing, China, 2005‐2024.

	Year, n (%)																			
Region	2005	2006	2007	2008	2009	2010	2011	2012	2013	2014	2015	2016	2017	2018	2019	2020	2021	2022	2023	2024
Baoding Town	16 (1.6)	16 (2.3)	18 (2.2)	23 (3.1)	25 (3.8)	19 (3.0)	24 (4.7)	22 (3.7)	21 (3.6)	11 (2.0)	13 (2.4)	14 (2.8)	4 (1.1)	9 (2.5)	9 (2.6)	7 (2.5)	5 (1.6)	5 (2.0)	5 (1.9)	8 (3.4)
Baoxing Town	34 (3.4)	34 (4.9)	40 (4.9)	22 (2.9)	34 (5.1)	21 (3.3)	16 (3.1)	18 (3.0)	20 (3.4)	20 (3.6)	19 (3.6)	9 (1.8)	13 (3.7)	8 (2.2)	17 (4.9)	11 (3.9)	14 (4.6)	13 (5.1)	10 (3.8)	2 (0.9)
Gaoping Town	7 (0.7)	5 (0.7)	6 (0.7)	6 (0.8)	7 (1.1)	8 (1.3)	10 (2.0)	6 (1.0)	8 (1.4)	14 (2.5)	8 (1.5)	11 (2.2)	8 (2.3)	9 (2.5)	6 (1.7)	3 (1.1)	3 (1.0)	6 (2.4)	7 (2.6)	2 (0.9)
Gaosheng Town	22 (2.2)	18 (2.6)	12 (1.5)	8 (1.1)	14 (2.1)	11 (1.7)	11 (2.2)	17 (2 9)	11 (1.9)	10 (1.8)	5 (0.9)	6 (1.6)	8 (2.3)	2 (0.6)	4 (1.2)	4 (1.4)	3 (1.0)	1 (0.4)	3 (1.1)	1 (0.4)
Gulong Town	7 (0.7)	8 (1.1)	18 (2.2)	11 (1.5)	8 (1.2)	11 (1.7)	4 (0.8)	9 (1.5)	6 (1.0)	4 (0.7)	2 (0.4)	2 (0.4)	3 (0.9)	2 (0.6)	3 (0.9)	1 (0.4)	1 (0.3)	1 (0.4)	4 (1.5)	2 (0.9)
Guoliang Town	32 (3.2)	12 (1.7)	18 (2.2)	11 (1.5)	15 (2.3)	15 (2.4)	9 (1.8)	17 (2.9)	13 (2.2)	7 (1.3)	17 (3.2)	12 (2.4)	6 (1.7)	3 (0.8)	3 (0.9)	3 (1.1)	5 (1.6)	5 (2.0)	3 (1.1)	3 (1.3)
Huilong Town	26 (2.6)	17 (2.4)	28 (3.5)	10 (1.3)	15 (2.3)	14 (2.2)	15 (3.0)	12 (2.0)	14 (2.4)	11 (2.0)	10 (1.9)	18 (3.5)	11 (3.1)	8 (2.2)	9 (2.6)	3 (1.1)	10 (3.3)	5 (2.0)	5 (1.9)	2 (0.9)
Jijia Town	17 (1.7)	13 (1.9)	15 (1.9)	11 (1.5)	12 (1.8)	11 (1.7)	9 (1.8)	13 (2.2)	5 (0.9)	5 (0.9)	9 (1.7)	8 (1.6)	5 (1.4)	5 (1.4)	7 (2.0)	5 (1.8)	9 (3.0)	1 (0.4)	3 (1.1)	6 (2.6)
Jinshan Town	36 (3.6)	19 (2.7)	18 (2.2)	23 (3.1)	20 (3.0)	13 (2.1)	14 (2.8)	14 (2.4)	14 (2.4)	20 (3.6)	17 (3.2)	14 (2.8)	6 (1.7)	7 (2.0)	10 (2.9)	7 (2.5)	8 (2.6)	2 (0.8)	5 (1.9)	1 (0.4)
Longgang Subdistrict	94 (9.4)	55 (7.8)	64 (7.9)	69 (9.2)	60 (9.0)	59 (9.4)	59 (11.6)	51 (8.6)	52 (8.9)	46 (8.3)	36 (6.7)	37 (7.3)	29 (8.3)	50 (14.0)	42 (12.1)	27 (9.5)	35 (11.5)	28 (11.0)	23 (8.6)	17 (7.3)
Longshi Town	6 (0.6)	11 (1.6)	8(1.0)	6 (0.8)	8 (1.2)	12 (1.9)	4 (0.8)	5 (0.8)	11 (1.9)	12 (2.2)	6 (1.1)	12 (2.4)	6 (1.7)	5 (1.4)	3 (0.9)	3 (1.1)	6 (2.0)	2 (0.8)	2 (0.8)	2 (0.9)
Longshui Town	115 (11.5)	83 (11.8)	112 (13.8)	88 (11.8)	89 (13.4)	95 (15.1)	47 (9.3)	75 (12.6)	85 (14.5)	73 (13.2)	69 (12.9)	69 (13.6)	61 (17.4)	50 (14.0)	36 (10.4)	32 (11.2)	40 (13.1)	46 (18.0)	39 (14.7)	35 (15.0)
Longtanzi Subdistrict	9 (0.9)	5 (0.7)	8 (1.0)	8 (1.1)	0	3 (0.5)	3 (0.6)	3 (0.5)	0	4 (0.7)	4 (0.7)	4 (0.8)	1 (0.3)	4 (1.1)	3 (0.9)	2 (0.7)	2 (0.7)	4 (1.6)	1 (0.4)	4 (1.7)
Sanqu Town	51 (5.1)	38 (5.4)	43 (5.3)	43 (5.8)	31 (4.7)	28 (4.4)	18 (3.5)	28 (4.7)	28 (4.8)	34 (6.1)	33 (6.2)	21 (4.1)	20 (5.7)	14 (3.9)	11 (3.2)	15 (5.3)	11 (3.6)	10 (3.9)	8 (3.0)	10 (4.3)
Shima Town	39 (3.9)	28 (4.0)	35 (4.3)	24 (3.2)	21 (3.2)	20 (3.2)	19 (3.7)	30 (5.1)	26 (4.4)	23 (4.2)	20 (3.7)	14 (2.8)	10 (2.8)	13 (3.6)	12 (3.5)	5 (1.8)	9 (3.0)	5 (2.0)	13 (4.9)	4 (1.7)
Shiwan Town	28 (2.8)	22 (3.1)	26 (3.2)	23 (3.1)	20 (3.0)	29 (4.6)	16 (3.1)	14 (2.4)	15 (2.6)	19 (3.4)	20 (3.7)	12 (2.4)	7 (2.0)	4 (1.1)	12 (3.5)	7 (2.5)	6 (2.0)	4 (1.6)	3 (1.1)	3 (1.3)
Shuanglu Subdistrict	15 (1.5)	8 (1.1)	11 (1.4)	10 (1.3)	10 (1.5)	12 (1.9)	7 (1.4)	10 (1.7)	17 (2.9)	25 (4.5)	19 (3.6)	11 (2.2)	6 (1.7)	13 (3.6)	13 (3.7)	18 (6.3)	17 (5.6)	7 (2.7)	8 (3.0)	13 (5.6)
Tangxiang Subdistrict	70 (7.0)	21 (3.0)	37 (4.6)	48 (6.4)	37 (5.6)	30 (4.8)	36 (7.1)	26 (4.4)	41 (7.0)	45 (8.1)	39 (7.3)	34 (6.7)	32 (9.1)	44 (12.3)	47 (13.5)	49 (17.2)	46 (15.1)	38 (14.9)	44 (16.5)	47 (20.1)
Tieshan Town	24 (2.4)	20 (2.9)	12 (1.5)	16 (2.1)	14 (2.1)	10 (1.6)	14 (2.8)	19 (3.2)	16 (2.7)	13 (2.3)	9 (1.7)	17 (3.3)	6 (1.7)	5 (1.4)	3 (0.9)	8 (2.8)	4 (1.3)	5 (2.0)	6 (2.3)	6 (2.6)
Tongqiao Subdistrict	5 (0.5)	4 (0.6)	7 (0.9)	6 (0.8)	6 (0.9)	6 (1.0)	5 (1.0)	13 (2.2)	4 (0.7)	5 (0.9)	3 (0.6)	5 (1.0)	4 (1.1)	8 (2.2)	6 (1.7)	8 (2.8)	7 (2.3)	4 (1.6)	2 (0.8)	5 (2.1)
Wangu Town	50 (5.0)	46 (6.6)	42 (5.2)	50 (6.7)	30 (4.5)	32 (5.1)	30 (5.9)	34 (5.7)	27 (4.6)	24 (4.3)	26 (4.9)	17 (3.3)	15 (4.3)	13 (3.6)	20 (5.8)	11 (3.9)	10 (3.3)	15 (5.9)	17 (6.4)	7 (3.0)
Yongxi Town	19 (1.9)	21 (3.0)	24 (3.0)	21 (2.8)	17 (2.6)	17 (2.7)	15 (3.0)	27 (4.5)	13 (2.2)	11 (2.0)	13 (2.4)	15 (3.0)	12 (3.4)	10 (2.8)	6 (1.7)	3 (1.1)	3 (1.0)	8 (3.1)	10 (3.8)	5 (2.1)
Youting Town	55 (5.5)	38 (5.4)	44 (5.4)	50 (6.7)	33 (5.0)	30 (4.8)	28 (5.5)	27 (4.5)	40 (6.8)	29 (5.2)	38 (7.1)	37 (7.3)	20 (5.7)	13 (3.6)	10 (2.9)	12 (4.2)	13 (4.3)	8 (3.1)	7 (2.6)	11 (4.7)
Yulong Town	23 (2.3)	14 (2.0)	15 (1.9)	21 (2.8)	24 (3.6)	16 (2.5)	18 (3.5)	17 (2.9)	14 (2.4)	12 (2.2)	16 (3.0)	18 (3.5)	4 (1.1)	8 (2.2)	8 (2.3)	7 (2.5)	3 (1.0)	2 (0.8)	6 (2.3)	5 (2.1)
Zhifeng Subdistrict	71 (7.1)	37 (5.3)	33 (4.1)	30 (4.0)	36 (5.4)	26 (4.1)	20 (3.9)	23 (3.9)	28 (4.8)	27 (4.9)	25 (4.7)	23 (4.5)	13 (3.7)	10 (2.8)	14 (4.0)	10 (3.5)	9 (3.0)	12 (4.7)	10 (3.8)	5 (2.1)
Zhong’ao Town	84 (8.4)	62 (8.8)	63 (7.8)	63 (8.4)	38 (5.7)	45 (7.1)	24 (4.7)	35 (5.9)	27 (4.6)	22 (4.0)	27 (5.1)	34 (6.7)	19 (5.4)	13 (3.6)	16 (4.6)	13 (4.6)	13 (4.3)	11 (4.3)	12 (4.5)	14 (6.0)
*Zhuxi Town*	45 (4.5)	46 (6.6)	53 (6.5)	46 (6.2)	39 (5.9)	37 (5.9)	33 (6.5)	29 (4.9)	31 (5.3)	28 (5.1)	31 (5.8)	34 (6.7)	22 (6.3)	27 (7.6)	17 (4.9)	11 (3.9)	13 (4.3)	7 (2.7)	10 (3.8)	14 (6.0)

**Figure 6.**
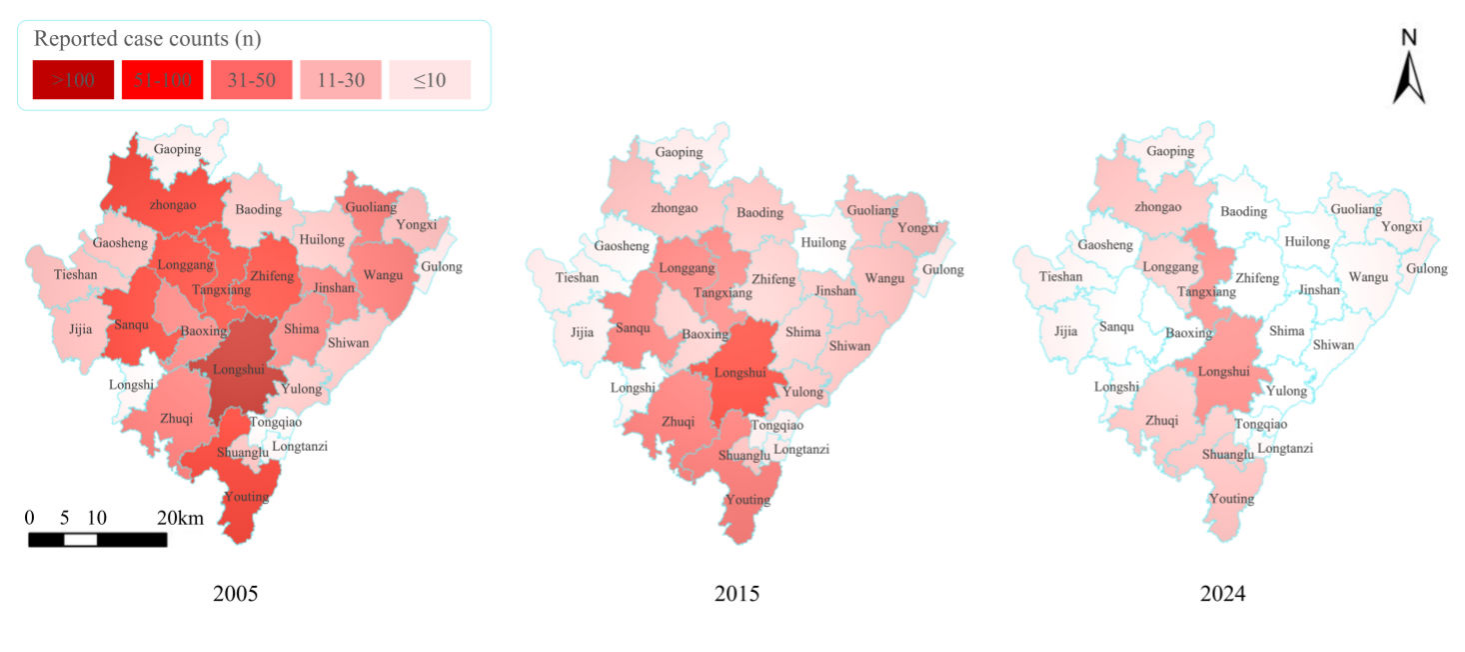
Spatiotemporal evolution of reported tuberculosis cases: Geographic heatmap across town/subdistrict in Dazu District, 2005‐2024.

We selected annual reported cases from 2005, 2015, and the most recent year (2024) to create geographical heatmaps (Figure 6). As shown, in 2005, Dazu District exhibited widespread high-intensity clustering of cases (>50 cases), with 7 towns and streets along the central axis (including Longshui, Longgang, and Tangxiang) each reporting over 50 cases. By 2015, the TB burden had decreased significantly across the region. Only Longshui Town remained a high-intensity cluster, while the other 6 areas had declined to medium (31‐50 cases) or medium-low intensity (11‐30 cases). By 2024, except for Tangxiang Street and Longshui Town, which were classified as medium-intensity areas, all other towns and streets fell into medium-low or low-intensity categories, of which 20 are low-intensity areas (<10 cases), with a coverage rate of more than 74% (Figure 6).

**Figure 7.**
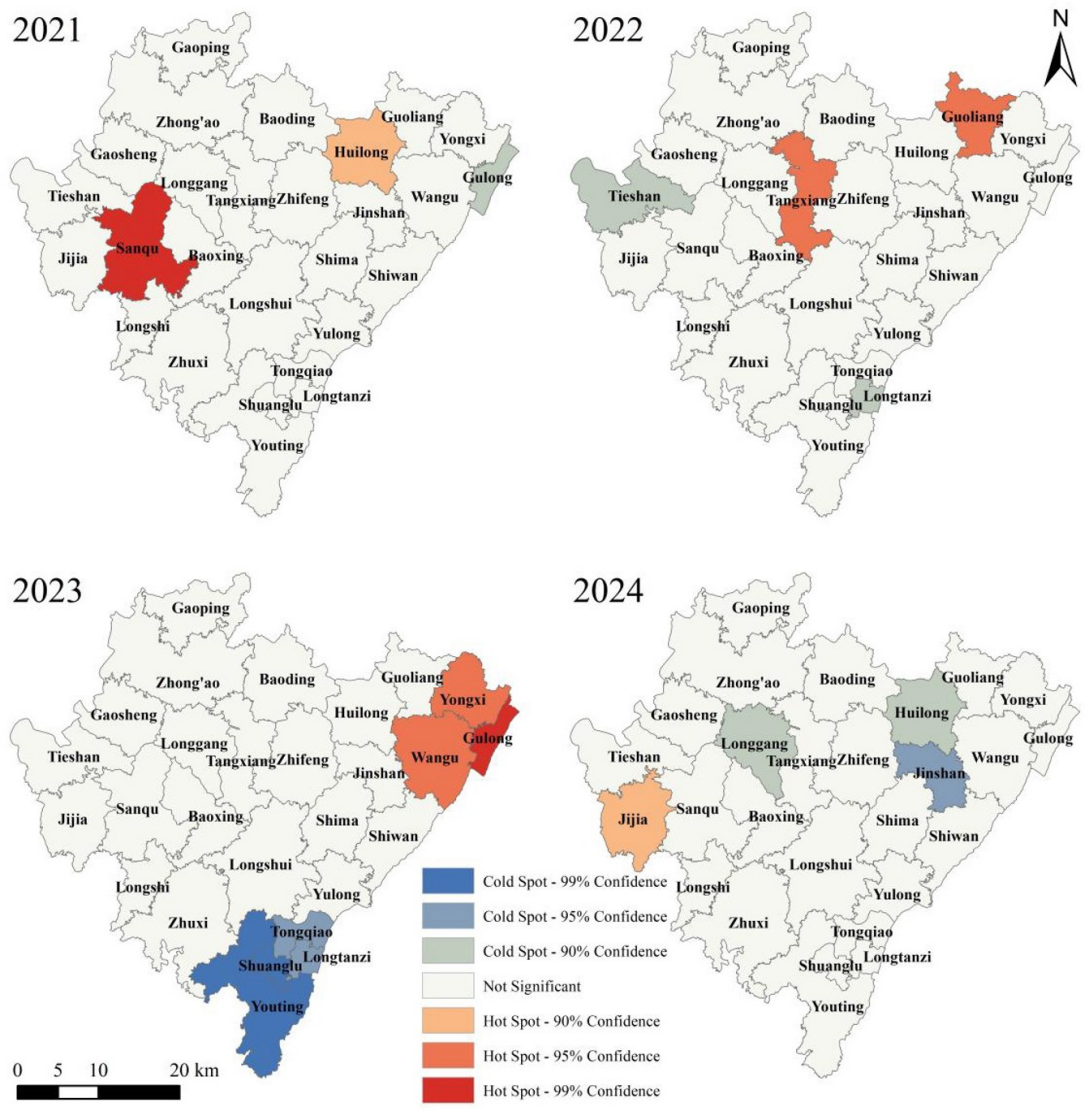
Local spatial autocorrelation analysis of pulmonary tuberculosis incidence in Dazu District, Chongqing, 2021‐2024.

We calculated the notification incidence rate for each township and subdistrict in Dazu District from 2021 to 2024, using the permanent resident population data from the 2020 Seventh National Population Census of China as the denominator, and subsequently performed a spatial autocorrelation analysis. The global spatial autocorrelation analysis (Table 6) indicated that the spatial clustering of township-level PTB incidence was not statistically significant overall during the 2021‐2024 period, with the exception of the year 2023, which exhibited significant spatial clustering rather than a random distribution (*P*<.001). Local spatial autocorrelation analysis (Figure 7) revealed indistinct spatial segregation of cold-spot or hot-spot areas for PTB incidence among the townships and subdistricts of Dazu District, with the identified cold-spot and hot-spot areas varying across different years.

**Table 6.** Global spatial autocorrelation analysis of pulmonary tuberculosis (PTB) incidence in Dazu District, Chongqing 2021‐2024.

Year	Moran *I*	*Z* value	*P* value
2021	0.232	1.44	.15
2022	0.197	1.28	.20
2023	0.669	3.831	<.001
2024	0.017	0.292	.77

### Univariate Analysis of Treatment Outcomes

Among 10,236 reported cases, excluding 159 cases still under treatment, a total of 10,077 cases were included in the treatment outcome analysis. There were 9203 successfully treated cases, yielding a treatment success rate of 91.3% (9203/10,077). Univariate analysis revealed that age (*χ*²_3_=78.265, *P*<.001), occupation (*χ*²_3_=10.228, *P*=.02), patient origin (*χ*²_5_=11.59, *P*=.04), treatment classification (initial treatment vs retreatment, *χ*²_1_=10.205, *P*=.001), diagnostic results (bacteriologically negative vs positive, *χ*²_1_=52.723, *P*<.001), HIV coinfection status (*χ*²_1_=14.724, *P*<.001), and treatment management modality (intensive phase supervision vs full-course supervision, *χ*²_2_=430.013, *P*<.001) were risk factors influencing treatment outcomes (Table 7).

**Table 7.** Univariate analysis of treatment outcomes among patients with pulmonary tuberculosis (PTB) in Dazu District, Chongqing, China, 2005‐2024.

Variable	Outcome		Chi-square (*df*)	*P* value
	Treatment success (n)	Unfavorable outcome (n)		
Gender			0.098 (1)	.75
Male	7186	687		
Female	2017	187		
Age (y)			78.265 (3)	<.001
<25	1414	110		
25‐44	2375	183		
45‐64	3642	303		
≥65	1772	278		
Occupation			10.228 (3)	.02
Farmers	7135	701		
Homemakers and unemployed	526	31		
Students	493	36		
Others	1049	106		
Case source			11.59 (5)	.04
Direct hospital visits	3291	301		
Referral	3408	346		
Tracking	1417	108		
Recommendation	937	97		
Health checkup	112	15		
Active screening	38	7		
Diagnostic delay			1.199 (2)	.55
≤28 days	2582	254		
29 days to 3 months	5570	513		
≥3 months	1051	107		
Treatment classification			10.205 (1)	<.001
Initial treatment	8676	800		
Retreatment	527	74		
Diagnostic results			52.723 (1)	<.001
Bacteriologically positive	3831	253		
Bacteriologically negative	5372	621		
Primary-level management			3.498 (1)	.06
Yes	5808	580		
No	3395	294		
HIV coinfection			14.724 (1)	<.001
Yes	218	40		
No	8985	834		
Comorbid diabetes mellitus			0.83 (1)	.36
Yes	254	19		
No	8949	855		
Treatment management modality			430.013 (2)	<.001
Intensive phase supervision	4054	668		
Full-course supervision	5149	206		
Complicated by extrapulmonary tuberculosis			0.243 (1)	.62
Yes	440	38		
No	8763	836		

### Multivariate Analysis of Treatment Outcomes

Collinearity analysis was performed for all variables. All variables showed tolerance >0.1, variance inflation factor <10, eigenvalues substantially greater than 0, and condition indices <30, indicating no significant multicollinearity issues. Using treatment outcome as the dependent variable (1=Treatment success, 0=Unfavorable treatment outcome), variables showing significance in univariate analysis were included as independent variables in multivariate binary logistic regression with the enter method. Multivariate logistic regression revealed that age, treatment classification, HIV coinfection status, bacteriologic test results, and treatment management modality were significant determinants of treatment success (Table 8).

**Table 8.** Binary multivariate logistic regression analysis of treatment outcomes.

Variable	*B*	SE	Wald chi-square *(df)*	*P* value	Exp (*B*)	95% CI of Exp (*B*)	
						Lower limit	Upper limit
Age (y)							
<25	—^a^	—	56.590 (3)	<.001	—	—	—
25‐44	0.562	0.145	15.012 (1)	<.001	1.755	1.320	2.332
45‐64	0.680	0.106	41.055 (1)	<.001	1.974	1.603	2.431
≥65	0.598	0.092	41.860 (1)	<.001	0.819	1.518	2.181
Treatment classification (retreatment, initial)	1.331	0.207	41.375 (1)	<.001	3.786	2.524	5.680
Bacteriologic test results (positive, negative)	1.044	0.127	67.293 (1)	<.001	2.841	2.214	3.646
HIV test results (positive, negative)	0.916	0.192	22.655 (1)	<.001	2.499	1.714	3.643
Treatment management modality (intensive phase supervision)	—	—	343.845 (2)	<.001	—	—	—
Full-course supervision	2.042	0.272	56.275 (1)	<.001	7.705	4.520	13.137
Constant	−2.795	0.594	22.140 (1)	<.001	0.061	—	—

a not applicable.

## Discussion

Our analysis of 2 decades of surveillance data yielded 3 main findings that align with the study objectives. First, Joinpoint regression confirmed a significant decline in the incidence of PTB (AAPC=–6.81; 95% CI –7.25 to –6.30%; *P*<.0001), with bacteriological positivity rates following a U-shaped trend over the study period. Second, spatial analysis revealed persistent high-burden areas, with Longshui Town and Tangxiang Subdistrict exhibiting the highest disease burden. Third, treatment success was significantly associated with younger age, initial treatment, HIV-negative status, bacteriologically negative results, and full-course supervised management.

Accurate data on TB incidence are difficult to obtain; consequently, reported incident case numbers serve as crucial indicators of disease burden and epidemiologic trends. From 2005 to 2024, a total of 10,236 patients with PTB were reported in our district. The annual notification incidence rate decreased from an initial rate of 131.2/100,000 population to 28.4/100,000 population in 2024, with an average annual rate of 65.2/100,000 population. This rate is slightly higher than the national average (60.77/100,000 population) [4] and the average annual notification incidence rate of Chongqing (54.6/100,000 population) [17] but lower than that of the Western China region (87.35/100,000 population) [4]. The notification incidence demonstrated an overall downward trend with an average annual decline rate of 7.7%, comparable to the annual decline rate observed in Chengdu City, China (7.75%) [18]. From an annual perspective, the TB case count in our district was particularly high in 2005, reaching 1000 cases, or 9.8% of the total cases reported during the entire study period. This historic peak is attributed to the full implementation of the Directly Observed Therapy-Short-course strategy [19] and improvements of the infectious disease reporting system that year. However, with the refinement of PTB prevention and control measures, annual case counts have gradually decreased, dropping to only 234 cases in 2024. The bacteriological positivity rate in our district initially decreased from 46.9% to 24.4% in 2016 and then gradually increased to 81.6% by 2024. This trend aligns with national reports on positivity rate changes [4], reflecting that our district’s epidemic prevention and control strategies have kept pace with national efforts. The increase is also associated with the widespread adoption of molecular biology detection technologies in recent years, particularly second-generation gene sequencing of bronchoscopic alveolar lavage fluid, which has significantly enhanced the bacteriological positivity rate of suspected PTB cases [20].

The incidence of PTB exhibits seasonal patterns [21], with the highest numbers of reported cases in our district occurring in March, predominantly concentrated during spring and summer, while winter case counts remained relatively lower. This phenomenon aligns with the findings of an international study [22]. Potential contributing factors include (1) cold winter weather increases indoor congregation with poor ventilation, facilitating *MTB* transmission; (2) reduced sunlight exposure during the winter decreases vitamin D synthesis; low vitamin D levels may increase susceptibility to TB [23]; (3) January and February coincide with China’s Spring Festival period. As TB symptoms often present atypically during early disease, many patients delay medical consultations until post-festival periods, leading to a surge in reported cases by March.

Our finding that cases were predominantly among male patients (male-to-female ratio of 3.57:1) was consistent with the findings of domestic and international studies [24,25]. This phenomenon may be attributed to gender-specific differences in hormone levels and genetic polymorphisms that influence susceptibility to *MTB* [26]. Many studies in humans and experimental animals have established clear links between sex-specific factors and the differential susceptibility of male and female individuals to a number of infectious diseases. Sex-specific determinants of Semin Immunopathol immunity include effects of sex steroid hormones as well as sex chromosome–encoded genes and microRNA [27]. Additionally, male patients often face systemic disadvantages in seeking or accessing TB care across various social contexts [28]. These findings highlight the necessity of incorporating gender-sensitive approaches into PTB prevention and control measures, particularly by enhancing diagnostic accessibility and screening services for male populations, to ensure equitable health care delivery across genders. This study revealed that reported PTB cases, in both male and female patients, were predominantly concentrated in the 15‐ to 64-year-old age group, consistent with previous research [29]. Notably, the proportion of cases among children less than 15 years of age is gradually decreasing in our district, while reported cases among those over the age of 65 years are progressively increasing. This trend suggests that our district must place greater emphasis on TB prevention and control in the older population. The observed pattern may be attributed to accelerated population aging, declining physiological function, and higher comorbidity rates among older adults, increasing susceptibility to TB. Conversely, the gradual reduction in cases among children under 15 years of age likely results from protection conferred by Bacillus Calmette Guerin vaccination and enhanced TB control measures targeting student populations. These findings highlight the necessity to increase the frequency of PTB screening among the older adults, particularly among male seniors, to reduce incidence rates in this vulnerable demographic.

Our finding that farmers constituted the majority (77.6%, 7948/10,236) of reported cases in our district is consistent with the findings of a previous study [30]. Potential explanations for this pattern include the following: (1) China’s status as an agricultural nation with a large rural population and high demographic proportion; (2) rural areas may experience relatively underdeveloped economic conditions, limited access to medical services, and inadequate prevention and control measures; (3) a generally lower educational status and limited awareness of PTB-related knowledge among farmers. TB-related knowledge, educational levels, and awareness of transmission risk factors correlate with disease incidence [31,32]. Therefore, enhanced education regarding PTB prevention and control measures is essential in rural communities.

Our observation of the second-highest case count (5.6%, 570/10,236) among homemakers and unemployed may reflect an association between relatively low income levels and unwillingness to seek medical care when symptoms emerge, which may facilitate TB transmission [33]. The third-highest case count (5.2%, 532/10,236) among students may be related to school population densities, intense academic pressure, and sleep deprivation [34] that contribute to both *MTB* transmission and increased susceptibility to post-infection disease onset and progression. These findings collectively highlight the necessity for targeted prevention and control measures focused on these high-risk populations in our district.

Significant geographical disparities of incidence were observed across towns and subdistricts. The highest reported case count (13.1%, 1339/10,236) observed in Longshui Town, followed by Longgang Subdistrict and Tangxiang Subdistrict, may be related to the large population bases of these 3 towns and subdistricts. Guoliang Town exhibited the highest average annual incidence rate (314.4/100,000), while Tongqiao Subdistrict showed the lowest (16.8/100,000). A total of 21 towns or subdistricts had incidence rates exceeding 100/100,000, highlighting the persistently severe epidemic in our district. This necessitates intensified prevention and control measures in these areas, with particular attention required to mitigate future epidemic trends in Guoliang Town. Global spatial autocorrelation analysis of township-level TB incidence in Dazu District (2021‐2024) showed significant clustering only in 2023 (*P*<.001). Local spatial autocorrelation revealed indistinct hot-spot or cold-spot segregation. We speculate that spatial autocorrelation may have limited sensitivity at this fine scale due to low case counts leading to unstable rates and high geographical proximity with shared risk factors among adjacent townships. Creating geographic heatmaps using annual case counts may better illustrate spatial variations in the epidemic.

Our treatment success rate of 91.3% was slightly lower than reported in previous studies [35,36]. Multivariate analysis revealed that age, diagnostic criteria, management approaches, HIV coinfection status, and bacteriological test results were risk factors that influenced treatment success. Variations in economic status, external environmental exposures, and stress levels across different age groups, combined with age-related differences in immune function, contribute to the heterogeneous manifestations of PTB pathogenesis. Compromised immune function and chronic comorbidities among people over 65 years of age increase susceptibility to TB [37].

Positive bacteriologic test results indicate a higher bacterial load that may be more pathogenic and pose greater therapeutic challenges. Retreatment cases exhibited lower treatment success rates due to higher drug resistance rates, poor adherence, prolonged treatment duration, and more extensive lung pathology. Patients enrolled solely in intensive phase supervision programs may initially benefit from health care worker engagement that may facilitate more regular medication intake and heightened awareness of their condition. However, once the intensive phase concludes without continued supervision, patients may mistakenly believe they are cured of TB, resulting in reduced adherence, irregular medication intake, and ultimately unfavorable treatment outcomes. Individuals with HIV coinfection experience varying degrees of immune suppression, resulting in higher susceptibility to *MTB* infection, lower treatment success rates, and higher mortality rates [38,39].

This study has certain limitations. First, when analyzing annual incidence rates across towns and subdistricts, we were unable to obtain specific permanent resident population figures for corresponding years, and the proportion of reported cases in each town relative to the district’s total annual cases was used as a substitute for the annual incidence rate. Second, potentially relevant variables such as nutritional status, silicosis comorbidity, and specific extrapulmonary TB manifestations were not included in the analysis of treatment outcomes. Third, underreporting inherent to passive surveillance systems, particularly among asymptomatic cases, may lead to an underestimation of true incidence. Fourth, the evolution of diagnostic techniques (including the introduction of molecular assays like Xpert *MTB* or rifampicin) over the 20-year period may influence the temporal comparability of bacteriological results. Fifth, univariate analyses involved multiple comparisons without statistical adjustment (eg, Bonferroni), increasing the risk of type I errors; thus, multivariate results should be prioritized. Finally, the findings, derived from Dazu District—an urban-rural transition zone in western Chongqing with a high farmer proportion (77.6%) and aging trend—may be most applicable to agricultural regions in central and western China and should be generalized to metropolitan core areas with caution.

In conclusion, the incidence of PTB in our district gradually decreased. Special focus should be directed toward monitoring epidemic trends among farmers and male individuals. Concurrently, treatment protocols should be enhanced for patients with PTB across different age groups, those with HIV coinfection, individuals receiving only intensive phase supervision, patients undergoing retreatment, and bacteriologically confirmed cases. These measures will facilitate the reduction of PTB incidence and the improvement of treatment success rates for patients in our district. Future research could focus on high-burden towns, explore and construct an active screening model for TB suitable for the grassroots level, and deeply study the impact mechanism and intervention strategies of labor mobility on the cross-regional transmission of TB, so as to provide assistance for the formation of collaborative prevention and control strategies at the district and county scale.

## Supplementary material

10.2196/78564 Multimedia Appendix 1 Occupational distribution and case count percentages of reported pulmonary tuberculosis (PTB) cases in Dazu District, Chongqing, China, 2005-2024.

10.2196/78564 Checklist 1 STROBE checklist.

## Abbreviations

AAPC: average annual percent change
APC: annual percent change
*MTB*: *Mycobacterium tuberculosis*
PTB: pulmonary tuberculosis
TBIMS: Tuberculosis Management Information System
WHO: World Health Organization
