# Supplementary figures and images for: Epidemiology and Treatment Outcomes of Pulmonary Tuberculosis in Dazu District, Chongqing, China, 2005-2024: Surveillance Study
Source: JMIR Public Health Surveill. 2025 Nov 28;11:e78564. doi: 10.2196/78564 (PMC12669922; doi:10.2196/78564)

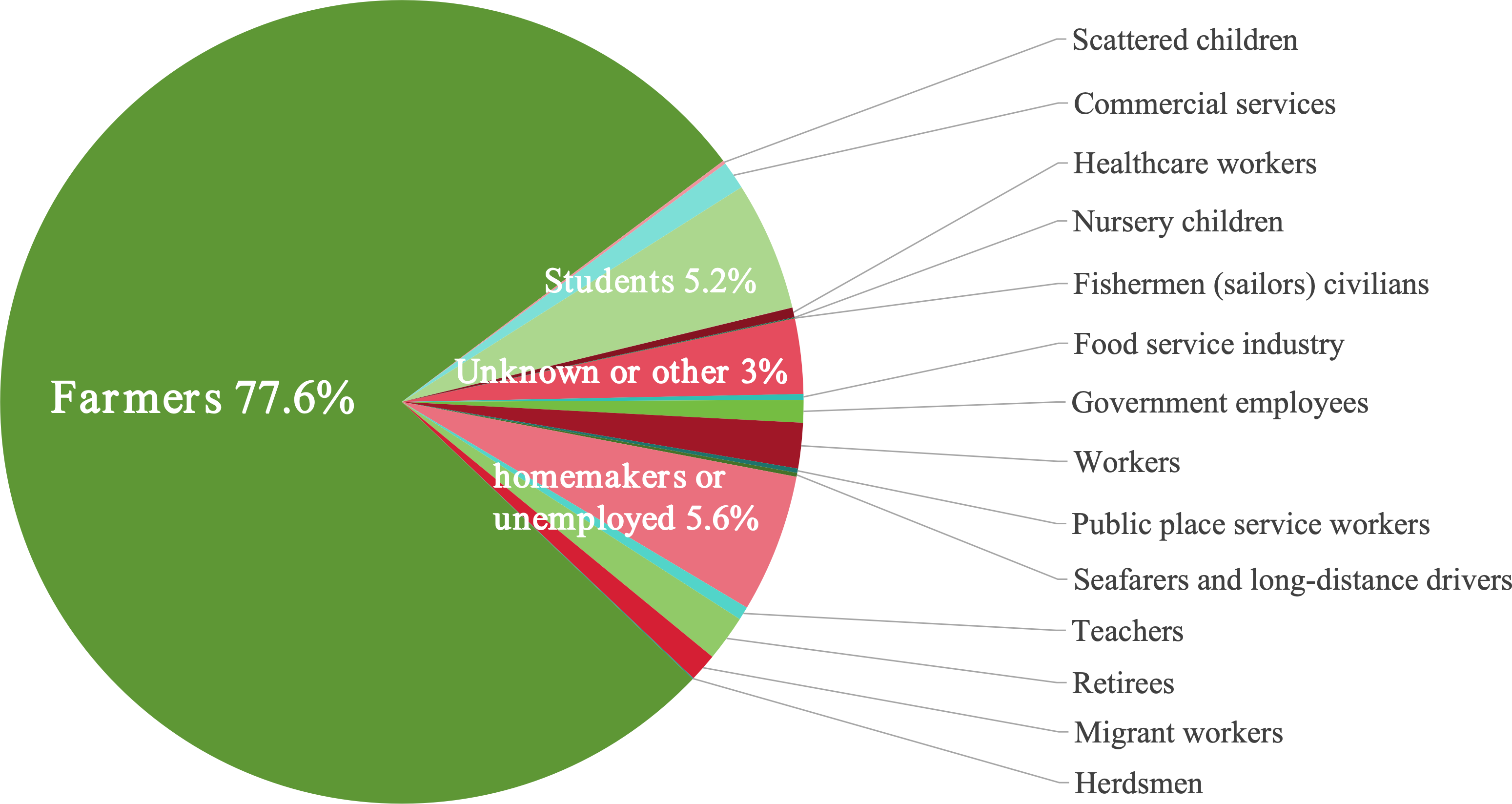

Supplement: Multimedia Appendix 1 [file publichealth-v11-e78564-s001.png]
